# Rationally designed microbial communities in agri-food production systems: from research to market

**DOI:** 10.1093/ismeco/ycaf121

**Published:** 2025-07-23

**Authors:** Dirkjan Schokker, Paul B Stege, Marie Duhamel, Martijn Bekker, Harro M Timmerman, Soumya K Kar, Hauke Smidt, Erwin G Zoetendal, Leo van Overbeek, Annelein Meisner

**Affiliations:** Wageningen Bioveterinary Research, Wageningen University and Research, Lelystad, Flevoland, 8221RA, the Netherlands; Wageningen Bioveterinary Research, Wageningen University and Research, Lelystad, Flevoland, 8221RA, the Netherlands; Wageningen Plant Research, Wageningen University and Research, Wageningen, Gelderland, 6708PE, the Netherlands; Wageningen Food and Biobased Research, Wageningen University and Research, Wageningen, Gelderland, 6700AA, the Netherlands; Wageningen Food and Biobased Research, Wageningen University and Research, Wageningen, Gelderland, 6700AA, the Netherlands; Wageningen Livestock Research, Animal Nutrition, Wageningen University and Research, Wageningen, Gelderland, 6708WD, the Netherland; Laboratory of Microbiology, Wageningen University and Research, Wageningen, Gelderland, 6708WE, the Netherlands; UNLOCK, Wageningen University and Research and Delft University of Technology, Wageningen (6708PB) and Delft (2629HS), the Netherlands; Laboratory of Microbiology, Wageningen University and Research, Wageningen, Gelderland, 6708WE, the Netherlands; Wageningen Plant Research, Wageningen University and Research, Wageningen, Gelderland, 6708PE, the Netherlands; Wageningen Plant Research, Wageningen University and Research, Wageningen, Gelderland, 6708PE, the Netherlands

**Keywords:** soil, soilless, rational designed microbial communities, synthetic communities, animal, chicken, primary production, genomics, crop, microbiome

## Abstract

Primary production needs to transition towards more sustainable systems that reduce environmental impact, mitigate climate change, and ensure healthy food production with limited use of chemical plant protection products, fertilizers, or antibiotics. Rationally designed microbial communities, or engineered microbial consortia, involve the intentional assembly of microorganisms that can underpin more sustainable primary production systems. Rationally designed microbial communities can for example, (i) enhance ecosystem resilience, (ii) improve bioremediation, (iii) enhance industrial processes, or (iv) prevent diseases. In the perspective, we discuss the route towards market applications with a focus on the methodology needed to rationally design microbial communities for applications in the agri-food production systems. Often *in silico* and *in vitro* approaches are considered as a continuous process that first consider the *in silico* genomic and then *in vitro* condition to develop microbial consortia. However, host–microbe interactions influence both the microbial community assembly and host phenotypes and need to be considered from an early stage when developing microbial communities. As such, we propose that the route towards market application(s) should, from a technical perspective include (i) the host of interest, (ii) a library of both slow and fast-growing species, and (iii) genomic information about functions present in the selected microbial consortia.

## Introduction

Rationally designed microbial communities (RDMCs), or engineered microbial consortia, involve the intentional assembly of microorganisms with specific functions and interactions [1–3]. RDMCs aim to modify microbial communities for desired outcomes, such as (i) enhanced ecosystem resilience, (ii) improved bioremediation, (iii) enhanced industrial processes, or (iv) disease prevention. RDMCs use existing knowledge of microbial interactions to design communities for specific goals, typically involving naturally occurring species for practical applications. In contrast, often the terminology “synthetic community” is used to indicate the engineering of microbial strains to focus on novel interactions and functionalities for both fundamental research and innovative applications. When these microbial products come to market, the terminology may hinder consumer acceptance [4, 5] and therefore should not be used. International agreements like the European “Green Deal” state that primary production should shift to more sustainable practices that reduce environmental impact, mitigate climate change, and ensure healthy and safe food production. Microbial communities can enhance host health, improve ecosystems functions, and suppress pathogens [6]. However, current agri-food systems still lack tailored microbiomes and primarily rely on chemicals for cleaning, sterilization, fertilization, and disease prevention (e.g. antibiotics and chemical plant protection products). These chemicals can have negative side effects, such as health risks and the spread of antibiotic and pesticide resistance genes [7]. Furthermore, they can accumulate in the environment, increasing water and air pollution [8]. The lag in adopting microbial inputs is due to their complexity, limited research, regulatory hurdles, and the entrenched use of chemical inputs in current agri-food systems [9, 10]. Addressing these challenges will require further research, investment, and regulatory support to unlock the full potential of microbial solutions.

While microbial communities in plants and animals differ, the underlying ecological principles are similar. Soilless ecosystems in greenhouse horticulture, such as hydroponics, often have low microbial diversity and lack a symbiotic (i.e. a close, often long-term relationship between two or more organisms, including mutualism, commensalism, or parasitism) microbiome at planting [11]. Similarly, newly hatched poultry have limited exposure to their parents, leading to compromised microbiome transfer and a microbiome not properly shaped by parental influence [12, 13]. The lack of a diverse and functional microbiome in soilless systems in greenhouses and young animals on livestock farms presents an opportunity to introduce defined microbial consortia that can establish themselves, that can successfully colonize, and influence community assembly, functioning, and resilience [14]. An RDMC can enhance resilience against (a)biotic stress and serve as a preventive measure, quickly filling ecological niches in young eukaryotic life stages with microbial communities that support the host.

Microbial community assembly is complex [15, 16]. Without an RDMC, stochasticity and dispersal affect how microorganisms arrive in new environments. Local physicochemical factors and available niches filter which strains can establish themselves. For instance, the first microbes to colonize the gut of livestock typically thrive in oxic conditions, creating an environment for anaerobes [17]. These early species influence the establishment of subsequent species, shaping the successional trajectories [18, 19] through facilitation, niche partitioning, direct inhibition, or niche pre-emption, and by altering the host’s physiological state [18]. The dynamics of these communities will depend on available niches and resources, including finding partners to supplement auxotrophies [20]. Adding RDMCs to ecosystems with few occupied niches can influence species establishment and promote a more stable microbiome [21]. A diverse microbial community may exhibit a broader range of functions [22], higher functional redundancy and adaptability to various environmental conditions [23], providing growth benefits and higher resilience against stress. The advantages of introducing microbial communities to disturbed ecosystems, due to biodiversity changes from stressors, have been demonstrated through soil transplantation for ecosystem restoration [24]. Similarly, fecal transplantation in chickens has balanced jejunal Th17/Treg cells and increased growth performance [25]. Thus, adding RDMCs could give beneficial microorganisms a priority advantage, enhancing resilience to future (a)biotic stresses [18]. However, designing a stable microbial community, where members work together to enhance functions like resilience and growth, remains a significant challenge. Designing RDMCs is challenging due to complex interactions, environmental variability, high microbial diversity, colonization and persistence issues, functional redundancy, and potential unintended consequences.

There are various approaches to designing RDMCs [6, 26]. In the top-down selection process, microorganisms are chosen from a complex microbial community based on ecology and evolutionary theories [27]. For instance, a reductive screening method identified a minimal microbial consortium necessary for specific functions, such as lignocellulose degradation [28]. In the bottom-up approach, microbial consortia are constructed using key stone species essential for specific ecosystem functions [26, 27], for example by focusing on a specific metabolism as a key driver of species interactions in microbial communities [22]. Genomics guided analysis (e.g. 29, 30) can help identify the necessary members of these consortia, potentially enhancing our ability to predict community assembly and function. A hybrid approach combines rational selection of candidate microorganisms through bioinformatics with the natural environmental and ecological conditions of the habitats of interest [26, 31]. This method allows for evaluation of candidate microorganisms’ interactions under various ecological conditions, such as low carbon conditions in soilless hydroponic systems during seedling growth [26, 31]. Interactions with the host can determine how well microorganisms colonize and persist within the host, how they compete or cooperate with native microbes, and how they contribute to the host’s health and resilience against stresses [32–35].

**Figure 1:**
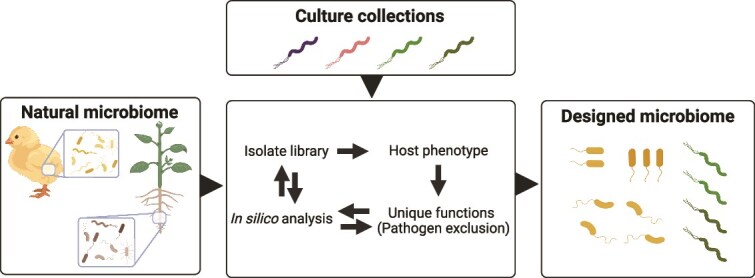
Schematic representation of the pathway toward market applications of RDMC. The process begins by acquiring the natural microbiome of interest, such as those associated with chickens or plants. This selection of the natural microbiome is a crucial step, as the associated microbes are the outcome of the host-environment interaction and will form the basis of the RDMC. Next, an in silico analysis is performed using metagenomic data to assess functional diversity, detect unwanted genes or pathogens, and develop an isolate library. The workflow then targets the desired host phenotype by identifying key functional traits and excluding pathogens. Finally, the designed microbial community—referred to as a rationally designed microbiome—is evaluated within the target ecosystem, with the potential to incorporate beneficial species from established culture collections. Created in https://BioRender.com.

This perspective explores pathways towards market applications, focusing on methodologies for the rational design of microbial communities in agri-food production systems using state-of-the-art technologies (Fig. 1). From acquiring natural microbiomes directly from their natural environment and performing *in silico* analyses to targeting desired host phenotypes and evaluating the designed microbiomes within their target ecosystems. We will discuss how to provide the conditions for strain selection, the inclusion of predictions based on genomics data, and host phenotypes in the selection process. This perspective focuses primarily on the initial strain selection process and does not delve into the complexities of optimizing consortium size or resilience.

### Conditions for rationally designed microbial communities

In an ideal setup, microbes that make up the RDMCs are identified via targeted isolation of microbes from target environments using a combination of genomic tools, cultivability of the selected strains, and the host phenotypic response (Fig. 1). In contrast, more untargeted alternative approaches, such as a shotgun culturomics wide selection, are typically expensive and laborious [36, 37]. In addition, using microbes available from culture collections and their standardized protocols ensures reliable and reproducible results, accelerating research. Do note, some approaches do not rely on isolating the consortia but instead focus on cultivating microbial consortia directly on farms. This method leverages the natural interactions and environmental conditions to enhance the effectiveness of the microbial communities in promoting plant growth and soil health [38]. Below we depict several conditions that are important for RDMC design and assembly.

### Rationally designed microbial communities assembly

Following the above, we suggest an RDMC design that combines the use of genomic tools, empirical knowledge about as many microbial strains as possible that were isolated from the host of interest, and the host phenotype response to those isolates (Fig. 1). The applied genomic tools use gene prediction, functional annotation, and sequence-based alignment tools, followed by core function determination to select representative bacterial species in the RDMC. Such core attributes include, e.g. for the microbe to maintain its role in the host, digestion, and competitive exclusion. It is further essential to assess the genomes from an in-house microbial collection with strains isolated from the environment of interest. The same *in silico* tools can then be applied again to determine the functionalities present per isolated strain. This would subsequently allow defining a consortium of strains that match the functions of the original microbiome and are able to grow under laboratory conditions. With such an approach one would allow mimicking the microbiome to the best extent that is currently feasible from a practical and industrial point of view. One must keep in mind that metagenomic assembled genomes are frequently far from complete and most functional capacities are predicted and lack experimental validation. While acknowledging that minimizing microbial consortia can lead to reduced functional diversity and redundancy, and increased vulnerability to environmental shifts, factors crucial for long-term RDMC stability.

### Strain collection

By using *in vitro* systems, such as rhizosphere microcosms [39–41] or Chicken Cecal ALIMEntary tRact Model (CALIMERO-2) [42], in the design of RDMCs, a strain collection can be selected with an associated database containing all metadata. While challenges such as genetic drift, metabolic dependencies, and the complexities of industrial scale-up are important considerations for RDMC development, these aspects fall outside the current scope of this perspective paper, which focuses primarily on the foundational principles of strain selection.

Such strain collection, from which the genome sequence has been determined, can be used in the development of the RDMCs for high throughput screening of culturable isolates for *in vivo* applications [43]. There are some conditions that need to be considered when developing the strain library for RDMCs *in vitro*: (i) a collection of strains should be available covering major functional capacities present in the ecosystem. However, this is not always the case, because of a lack of (selective) culturability of species and knowledge gaps of unknown species. As such, efforts should be made to improve culturability of strains. For example, resuscitation/awakening of dormant states of the viable but nonculturable cells [44] could improve success of microbial cultivation that are in these states. In addition, slow growing species may need specialized growth media that favor their growth in laboratory conditions [45]. Slow-growing microorganisms play critical roles in maintaining ecosystem function, particularly through processes like the degradation of complex organic matter and nutrient cycling; while fast-growing microbes may frequently act as opportunistic responders to changing conditions, slow growers—including ecologically essential fungi—often contribute foundational services such as disease suppression and long-term soil health. [46]. Although it will remain challenging to include slow-growing species in microbial consortia, this will increase the chance to develop stable consortia consisting of both slow-growing and fast-growing species. (ii) For creation of RDMCs, selecting community members that exert mutualistic interactions are desired. Mixing opportunistic pathogens, causing disease when host’s immune system is compromised, with those occupying similar niches may help identify strains best adapted to the system of interest. (iii) Microorganisms, including protists, archaea, viruses, and fungi, should be isolated from the system that they are intended to be used to increase success of establishing in that ecosystem. To include protists, archaea, viruses, and fungi in an RDMC, one can isolate them using selective media and conditions tailored to their unique requirements, and ensure environmental conditions mimic their natural habitats to enhance their establishment and functionality within the ecosystem. (iv) The *in vitro* conditions should mimic the *in vivo* conditions for which the RDMC is being developed. For example, microbial consortia for plants need oxic and C-limited conditions, whereas gut microbial consortia in animals need anoxic conditions rich in the carbon types present in the gut system of interest. However, mimicking the exact *in vivo* conditions is a huge challenge, especially when it concerns host-derived factors which are nearly impossible to mimic completely. For example, carbon sources from root exudates are dependent on plant size and are excreted continuously. An additional challenge will be matching *in vitro* requirements to support growth of all different microbes simultaneously, such as temperature, pH, trace elements and vitamins. *In vivo*, micro-niches can uncouple different strains. In the gut, for example, mucus-associated microbes will have a different niche than those occurring mostly in the lumen. In addition, from a practical point of view of RDMC production, microbes should not produce components that negatively affect the growth of others. (v) one should be able to store and mix all strains such that these can be used for inoculation of the desired niche. This practically means that all strains should show high survival during freezing and storage.

Given the potential for RDMC applications, product safety must be a paramount consideration throughout the strain collection process. To ensure responsible development and deployment, we will prioritize the inclusion of demonstrably non-pathogenic strains with well-characterized environmental safety profiles, minimizing any risk of unintended consequences upon introduction into target ecosystems.

### Selection of strains based on genomic data

*In silico* approaches can be used to identify functions in natural microbiomes and exclude pathogens or isolates with unwanted genomic information, such as transferable AMR genes [47]. Research suggests that probiotics change AMR genes in ways that depend on both the individual and the specific antibiotic [48]. Nevertheless probiotics, along with pre- and synbiotics are among the possible strategies to mitigate multi-drug resistance [49, 50]. Such information is needed to develop an RDMC that underpins the necessary functionality, without endangering the ecosystem. Existing approaches to select RDMCs *in silico* can roughly be divided into two groups: (i) tools that use the information present in metagenomic data to determine the metabolic functions [51–53] and (ii) tools that additionally require knowledge on the available substrates that are present in a system and the corresponding concentration [54]. In practice, the latter approach is commonly applied when the goal is to alter production of a metabolite or the activity of specific metabolic pathways. Bioinformatic tools like Minimal Microbial Consortia creation tools (MiMiC [54]) rely solely on metagenomic data without any prerequired knowledge on available substrates and the active state of the strains. Analysis starts by accessing the predicted genes present in the microbiome of each sample and which function these encode. Subsequently, the microbial genomes of the isolates are supplied and ranked by matching functions with those present in the natural microbiome. The microbes that contribute most in terms of the matching number of functions are prioritized, thereby resulting in an RDMC covering as many functions as possible that are present in a natural microbiome. With this approach the intention is to generate a stable consortium that supports (a)biotic resilience. Thus, MiMiC can select an RDMC based on putative functions in the metagenome but focuses on individual genes and not yet on gene pathways. In addition, the used approach should include analyses for the capability of microorganisms to interact and co-exist and exclude pathogens and unwanted genes. For example, tools have been developed for differentiating *Pseudomonas* species with plant growth promoting capacities from *Pseudomonas* species with pathogenicity to plants [55]. To this end it should be noted that there is additional strain-level variation, as it has been shown for example that different strains of *Pseudomonas syringae* can act as pathogen or probiotic [56]. Another feature to consider is co-operative and competitional potential of microbes, by calculating their overlapping metabolic profiles [57]. Current tools, however, do not implement the potential contributing or inhibiting effects on growth or reaction fluxes that are encoded in these metabolic profiles. In addition, these tools do not consider the excretion and role of secondary metabolites, while these secondary metabolites can play a key role in microbial interactions.

### Host phenotypes

A host is an additional active player in the ecosystem that needs to be considered, as a two-way interaction exists between microbes and their host. These interactions are also important in shaping the microbial community. For some hosts, it is not necessary for the RDMCs to persist. For example, when broiler chickens are colonized by an RDMC, the immediate beneficial effects on the host could be sufficient for that time-period, even if the RDMC does not persist long-term. In addition, hosts will constantly be colonized by microbes via the feed water and the environment. The RDMC will influence and steer also the microbial communities that disperse from the environment [58], even if members of the RDMC would be non-persisters [59]. Host responses to microbial consortia may pose selection pressure on the strains establishing in the host ecosystem and may influence community assembly trajectories of the microbial consortia. However, host–microbe interactions are not considered *in vitro* [60]. Often, one is interested in host responses to RDMCs, such as increased host growth rates, fitness, or resistance against pathogens. These host phenotypes are difficult to study with *in silico* or *in vitro* approaches only. An approach to develop minimum consortia is to focus on the intended phenotypic response of the host [61, 62]. By including host phenotypes in the selection process of the minimal consortia, the selection can focus on the phenotype of interest and the host influence on the microbial consortia. Specific phenotypic traits, such as immune responses, metabolic processes, and physical barriers, can influence which microbes are able to colonize and thrive within or on the host [63]. The follow-up research questions can focus on microbial functions, microbe-microbe interactions, microbe-host interactions and evolutionary mechanisms important for the host phenotypic response [64]. However, *in vivo* experiments may be challenging when meeting the ethical considerations of experiments with animals. Animal experiments should be mitigated by adhering to the Replacement, Reduction, Refinement (3R) principle (https://nc3rs.org.uk/). So, alternative approaches must be considered when including animal hosts and its characteristics into the selection process of RDMCs. Alternative phenotype systems include *in vitro* gastrointestinal models like the intestinal model, Simulator of Human Intestinal Microbial Ecosystem, or lab-on-a-chip systems and may provide tools to also mimic host-microbiome interactions. Such *in vitro* approaches can effectively simulate host-microbiome interactions, aiding in the selection of RDMCs for livestock, without harming animals.

**Figure 2:**
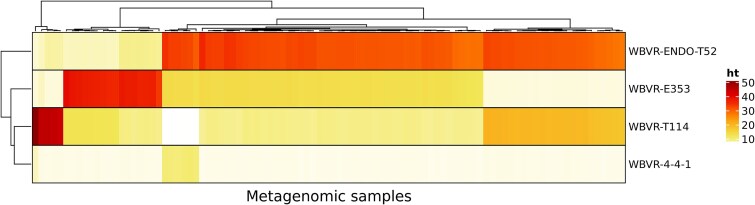
Heatmap of rationally designed microbial consortia explaining 60% of the functions present in a rhizosphere microbiome of tomato plants (from Oysermann et al. 2022 [65]). Each column represents a metagenomic rhizosphere sample, whereas rows represent four isolates used in this experiment. The color represents the percentage of gene functions a species can contribute to the microbial community.

### Recommended evaluation of minimal microbial consortia

**Figure 3:**
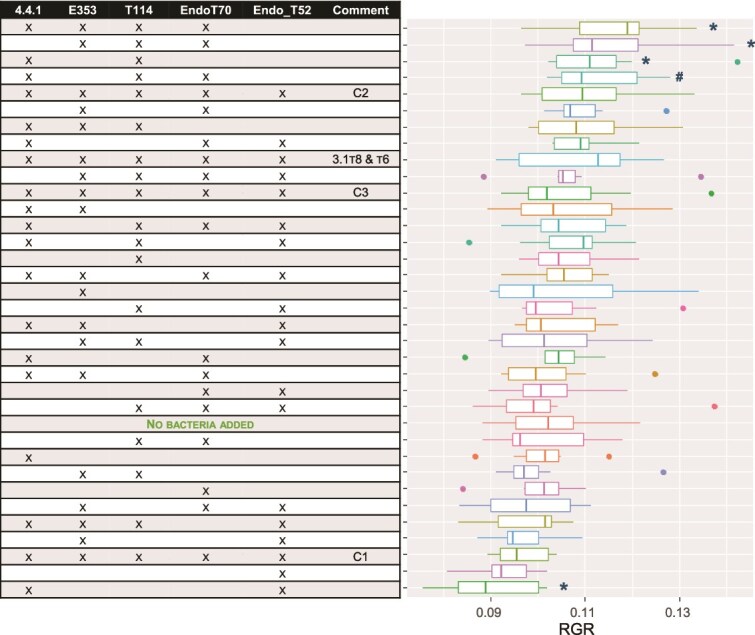
RGR of tomato plant seedlings. The names of the bacteria can be found in the supplemental information. The final concentration was 6.11e4 cells per plant. The five species mixtures were given in three concentrations: C1 received 6.11e3 cells per plant; C2 received 6.11e4 cells per plant and C3 received 6.11e5 cells per plant (C3). The bacterial consortia affected the RGR of the seedlings (F34, 213 = 1.88; *P* = .004). There were three bacterial consortia that increased the RGR of the seedlings in comparison with the control seedlings where bacteria were not added: [4.4.1, E353, T114, EndoT70], [E353 T114, and EndoT70], and [4.4.1 and T114]. In addition, bacterial consortium [4.4.1, T114, and EndoT70] tended to increase the RGRs in comparison to the no bacteria treatment. The consortium [4.4.1 and EndoT52] decreased the RGR in comparison to the control. Significance of consortia in comparison to the no bacterial control as evaluated with planned contrasts: ^*^P < .05; #*P* < .1.

To evaluate the described RDMC approach, we investigated which species combinations should be evaluated under *in vivo* conditions to be able to select the plant phenotype of interest. The plant phenotype was studied in the seedling phase where we aim for optimal growth of the seedlings. Thereto, a strain library isolated from tomato plants was established (see supplemental information), and the functions present in the metagenome of the isolates were compared with all functions present in the metagenome of a rhizosphere community of tomato plants [65]. A minimum of four isolates, namely *Pseudomonas chlororaphis subsp. aureofaciens* NBRC (4.4.1), *Herbaspirillum lusitanum* P6–12 (E353), *Pseudomonas entomophila* (T114), and *Arthrobacter* sp. EpRS66 (EndoT52), was found to cover 60% of the predicted functions present in the tomato rhizosphere microbiome (Fig. 2). This 60% indicates a substantial, though not complete, understanding of the community’s metabolic capabilities. Several studies have investigated rhizosphere microbial dynamics, demonstrating how factors like biochar amendments [66], bacteriophage introduction [67], nutrient limitation [68], and even the inherent composition of tomato rhizospheres [69], can influence community structure and stability. We validated this *in silico* result by adding different combinations of bacterial strains to germinated tomato seedlings (see supplement for details). As the bioinformatic tool excluded EndoT70, as this strain exhibited a similar, yet slightly smaller set of functions [based on protein families (Pfam)], namely 2074 functions compared to 2080 functions for strain EndoT52, we also included this strain. The *in vivo* experiment revealed a similar consortium of four bacterial species; 4.4.1, E353, T114, and EndoT70, that most strongly increased the relative growth rates (RGRs) of seedlings compared to the seedlings without bacteria added. Interestingly, adding single bacteria did not increase the RGR of seedlings compared to the seedlings without added bacteria (Fig. 3). The mixture selected by the *in silico* approach did not adversely affect plant seedlings. By combining both the host phenotype and the *in silico* approach, an RDMC could be built. From this experiment it could be concluded that RDMCs can significantly influence the RGR of tomato plant seedlings. Three consortia, [4.4.1, E353, T114, EndoT70], [E353, T114, EndoT70], and [4.4.1, T114], increased the growth rate compared to the control, while the consortium [4.4.1, EndoT52] decreased it. These findings indicate that specific bacterial mixtures can enhance plant growth.

### From research to market

The aim of this perspective is to discuss applications of RDMCs and product development in agri-food production systems by using current state-of-the-art technologies. We focus on the deployment of beneficial effects of early colonizers in ecosystems with low microbial diversity. This involves strategically introducing these microbes at optimal times, such as during planting or early life stages, to enhance microbial diversity and ecosystem resilience. Consequently, this approach aims to decrease the usage of plant protection products and antimicrobials in circular agri-food production. Often *in silico* and in vitro approaches are considered as a continuous process to develop microbial consortia with beneficial effects towards the host. However, host–microbe interactions influence both microbial community assembly and host phenotypes. Furthermore, while host phenotypes are the outcomes of interest of RDMCs designed for application in host-associated systems, they are often not included in *in silico* or in vitro approaches. As such, the route towards market application(s) should, from the technical perspective include: (i) the host of interest, (ii) a library of both slow and fast growing species that are culturable with knowledge about the pathogenicity of the isolates [e.g. by linking to known curated databases (http://www.mgc.ac.cn/VFs/)], and (iii) knowledge about functions present in the selected microbial consortia. Nevertheless, different approaches need to be considered for different agroecosystems, such as implementing machine learning and other AI-driven modeling for predicting microbial interactions, lab-on-a-chip for high-throughput screening, CRISPR-based genetic engineering for functional screening, or *in silico* approaches for animals and host phenotypic responses for plants. After the RDMC is defined from a technical perspective, efforts are needed to scale up the production and characterization of microbial strains and consortia. This will need an alternative approach than current production of beneficial strains, also focusing on non-spore formers and slow growing species in RDMCs. A more detailed discussion of terminology, including e.g. the possible differences and overlaps between RDMCs, SynComs, (multispecies) probiotics and synbiotics, is beyond the scope of the current review. In addition, product safety tests and registration of the RDMC need to be considered. Ensuring product safety is a critical aspect of moving RDMCs from research to market. By prioritizing non-pathogenic and environmentally safe microbes [70–73].

### Outlook

Our suggested approach may, currently, not be feasible for all ecosystems. Since it requires the microbial functions in the ecosystem or host to be known, or at least predictable, which is dependent on the completeness of public databases or genomic sequences of the microbiome of the niche in combination with the use of *in silico* tools and availability of experimental data. Additionally, it also relies on the ability to define the host’s optimal state. Performing *in vitro* experiments to validate the putative functionality will be essential for further implementation. The exploitation of upcoming microbiome-based innovations, such as RDMCs, needs to take into consideration the (existing) interactions with and between relevant stakeholders, such as farmers, citizens, or politicians. There is a lack of awareness of the benefits and potential of microbiomes among different stakeholder groups [74, 75]. In addition, the unpredictable and unreliable results of single strain applications on crops led to a loss of trust of the growers in biologicals, not considering them as suitable alternatives to chemicals [23, 76]. A better understanding of microbial consortia and the availability of “stable” RDMCs can help restore this trust. As such, interactions with different stakeholders to increase awareness of potential benefits of microbiomes and their efficacy are needed to increase the acceptance of future use of RDMCs for sustainable food production in the future.

The knowledge on microbial consortia, their development and deployment are increasing more rapidly than implementation of correct regulatory scientific assessment processes for microbial products in Europe [77, 78]. Current legislation focuses on the registration of individual consortium members, with current EU regulations being less supportive compared to those in the USA. This makes EU registration a more complex process, with low flexibility and high diversification among actors, and this EU registration process takes about 66 months compared to US registration approximately 26 months. Modernizations of existing policies, i.e. Common Agricultural Policy, is needed to transition towards a more circular primary production system. While the EU has recently made adjustments to facilitate the selection of consortia as viable ingredients, the overall efficiency of the registration process remains a topic of concern within the industry [79].

## Supplementary Material

Supplement_File_ycaf121

supplemental_data_plant_ycaf121

## Data Availability

Sequence data associated to the seven bacterial isolates for the plant experiment, namely *P. chlororaphis subsp. aureofaciens* NBRC (4.4.1), *H. lusitanum* P6–12 (E353), *Arthrobacter sp.* (EndoT70), *Arthrobacter sp.* EpRS66 (EndoT52), *Pseudomonas entomophila* (T114), *Lysobacter enzymogenes* (3.1 T8), and *Bacillus thuringiensis* serovar higo (T6), and accompanying data are available on ENA with accession number PRJEB87828. The shotgun metagenomic sequencing data of the rhizosphere microbiome in tomato, associated with PRJNA787039 and PRJNA789467, were retrieved from the NCBI database. The plant data set is available in the supplemental information. Associated scripts and pipelines are available on GitHub page: https://git.wur.nl/CVI_PathogenOmics/rational_designed_microbiome.
