# Supplementary material for: Rationally designed microbial communities in agri-food production systems: from research to market
Source: ISME Commun. 2025 Jul 23;5(1):ycaf121. doi: 10.1093/ismeco/ycaf121 (PMC12376040; doi:10.1093/ismeco/ycaf121)
Supplement: Supplement_File_ycaf121 [file supplement_file_ycaf121.pdf]

Supplemental information

Plant experiment

Preparation of bacterial consortia

Bacterial isolates were grown individually in the dark at 25°C for one week in the plant growth media (1). After one week, the number of bacterial cells per ml were determined using impedance flow cytometry and calculate the total amount of bacteria that needed to be added to the consortia mixtures. For each of the 34 conditions, bacterial consortia were developed by adding the number of bacterial cells to the tube up to a total of $1.22e^5$ bacterial cells per ml.

Execution of plant experiment and phenotypic measurements

In this experiment, the effect of an increasing number of bacterial species in the consortia was evaluated on plant seedling performance. Seeds of *Solanum Lycopersicum* root stock plants (Bayer) were sown into stonewool plugs on 13 October 2022. On 25 October, 241 germinated seedlings were transferred to stonewool blocks and $6.11e^4$ bacterial cells per plant were added to the blocks. Within WUR, our own strain library contained bacterial isolates that were isolated from greenhouse grown plants. All combinations (1-2-3-4-5 species) of the following 5-species mixtures were evaluated: *Pseudomonas chlororaphis subsp. aureofaciens* NBRC (4.4.1), *Herbaspirillum lusitanum* P6-12 (E353), *Arthrobacter sp.* (EndoT70), *Arthrobacter sp.* EpRS66 (EndoT52), *Pseudomonas entomophila* (T114) in seven replicates. There was also a 7-species mixture that contained in addition to the 5-species mixture the following strains: *Lysobacter enzymogenes* (3.1T8), *Bacillus thuringiensis* serovar higo (T6). In addition, one treatment did not have addition of isolates and was considered the control (reference) treatment. There were also two additional concentrations of the 5-species mixture, namely $6.11e^3$ cells per plant (C1) and $6.11e^5$ cells per plant (C3). This resulted in 34 conditions that were evaluated. The length of the seedlings was measured upon transfer of the seedlings to the stonewool blocks and two weeks after the addition of the bacterial consortia. The length measurements were used to calculate relative growth rate (RGR) of the seedlings as:

$$RGR = \frac{(\text{Ln}(\text{length T2}) - \text{Ln}(\text{length T1}))}{\text{Time (here 14 days)}}$$

Where T1 refers to time-point 1, and T2 refers to time-point 2. The plants received supplemental standard light during 12h (90% red, 5% blue and 5% green) per day and were grown under standard temperature conditions (22°C during the day and 15°C at night). The stonewool blocks received sterilized plant growth media that was prepared according to instructions provided (1).

Validation of potential functions of bacterial isolates

The validation of the potential functions of selected bacterial isolates in our strain library and the verification of the cumulative functions of the full microbiome genomes observed in the rhizosphere of tomato plants. A publicly available dataset was selected to determine an *in silico* designed minimal microbiome. This included 96 tomato plant rhizosphere metagenomic samples from the study of Oysermann *et al*. (2). The MiMiC release of 02-2021 was used to analyse these samples and determine a minimal consortium of ten bacterial species, which covers most microbial functions present in the metagenomic samples (3). In addition to metagenomic samples, MiMiC requires a genome database of bacterial species. The bacterial isolates were used as input for the genome database. Gene functions of the genome database and metagenomic samples were determined in parallel according to the MiMiC workflow. This includes assembly using metaSPAdes v3.15.4, contig size selection of 500bp using the reformat function of BBmap v39.01, gene detection and annotation using Prodigal v2.6.3, HMMER v3.3.2 and the protein family (PFAM) database release 16062022. Microbes that contributed most in terms of the matching number of functions present in the metagenomic samples together shaped a minimal consortium. KofamScan v1.3.0 (4) and the R pathview package v1.36.1 (5) were consequently used to translate the results into KEGG pathways. All steps of the workflow are listed on this github page: https://git.wur.nl/CVI_PathogenOmics/rational_designed_microbiome.

To analyze the RGR between the different treatments, statistical tests were performed in R (version 4.0.2 (6)), where a one-way ANOVA was performed by using bacterial isolates as fixed factor. Hereafter, treatment contrasts were performed where each treatment was tested against the control treatment, i.e. no added bacteria (7).

References

1. De Kreij C, Voogt W, Baas R. Nutrient solutions and water quality for soilless cultures. Applied Plant Research, Division Glasshouse; 2003.
2. Oyserman BO, Flores SS, Griffioen T, Pan X, van der Wijk E, Pronk L, et al. Disentangling the genetic basis of rhizosphere microbiome assembly in tomato. Nature communications. 2022;13(1):3228.
3. Kumar N, Hitch TCA, Haller D, Lagkouvardos I, Clavel T. MiMiC: a bioinformatic approach for generation of synthetic communities from metagenomes. Microbial biotechnology. 2021;14(4):1757-70.
4. Aramaki T, Blanc-Mathieu R, Endo H, Ohkubo K, Kanehisa M, Goto S, et al. KofamKOALA: KEGG Ortholog assignment based on profile HMM and adaptive score threshold. Bioinformatics. 2020;36(7):2251-2.
5. Luo W, Brouwer C. Pathview: an R/Bioconductor package for pathway-based data integration and visualization. Bioinformatics. 2013;29(14):1830-1.
6. R Core Team. R: A language and environment for statistical computing. R Foundation for Statistical Computing, Vienna, Austria. 2020.
7. Crawley MJ. The R book. West Sussex John Wiley & Sons, Ltd; 2013.
